# Histopathological Correlation of Atypical (C3) and Suspicious (C4) Categories in Fine Needle Aspiration Cytology of the Breast

**DOI:** 10.1155/2013/965498

**Published:** 2013-09-23

**Authors:** Prashant Goyal, Shelly Sehgal, Soumyesh Ghosh, Deepti Aggarwal, Pritesh Shukla, Awanindra Kumar, Ruchika Gupta, Sompal Singh

**Affiliations:** ^1^Department of Pathology, Swami Dayanand Hospital, Dilshad Garden, Delhi 110095, India; ^2^Department of Pathology, Hindu Rao Hospital, New Delhi 110007, India; ^3^Department of Pathology, Chacha Nehru Bal Chiktsalaya, New Delhi 110031, India

## Abstract

*Introduction.* According to the National Cancer Institute (NCI) guidelines in 1996, breast lesions are categorized as C1 to C5 on fine needle aspiration (FNA) cytology. Very few studies are available in the English literature analyzing histopathology outcome of C3 (atypical, probably benign) and C4 (suspicious, probably malignant) lesions. Our study aims to correlate FNA cytology of breast lump diagnosed as C3 and C4 lesion with histopathological examination. *Methods.* During a period of 2 years, 59 cases of C3 and 26 cases of C4 were retrieved from total 1093 cases of breast FNA. All the cases were reviewed by two cytopathologists independently. The final 24 cases of C3 and 16 cases of C4 categories were correlated with histopathological diagnosis. *Result.* Among C3 category, 37.5% revealed malignant findings, whereas of C4 category, 87.5% were malignant on histopathology. This difference was statistically significant (*P* = 0.0017). Sensitivity, specificity, positive predictive values, and negative predictive value of C4 category in diagnosing breast malignancy were 60.8%, 88.2%, 87.5%, and 62.5%, respectively. *Conclusion.* Although FNAC is simple, safe, cost-effective and accurate method for diagnosis of breast masses, one must be aware of its limitations particularly in C3 and C4 categories. Also, since both these categories carry different probabilities of malignancy and thus different management, we therefore, support maintaining C3 and C4 categories.

## 1. Introduction

Breast cancer is the most common malignant neoplasm affecting women worldwide. In India, breast cancer accounts for around 30% of all cancers in women. It has speedily replaced cervical cancer as the most common cancer among Indian women. According to figures from the National Cancer Registry, one in every 25 Indian women is likely to suffer from breast cancer at some point of time.

Fine needle aspiration (FNA) cytology has become widely accepted as a reliable diagnostic tool for diagnosing breast masses. It is a simple, quick, safe, less invasive, and less expensive method with high sensitivity and specificity. The diagnostic categories used in FNA cytology of the breast masses are inadequate (C1), benign (C2), atypical, probably benign (C3), suspicious of malignancy (C4), and malignant (C5) according to the National Cancer Institute (NCI) guidelines in 1996 [1]. However, Howell [2], and Kanhoush et al. [3] suggested the use of a single term, such as “equivocal”, to describe inconclusive (C3 & C4) diagnoses on breast FNA cytology.

Very few studies are available in the English literature analyzing histopathology outcome of C3 (atypical lesion) and C4 (suspicious lesion) lesions. Our study aims to correlate fine needle aspiration (FNA) cytology of breast lump diagnosed as C3 and C4 lesions with histopathological examination.

## 2. Material and Methods

This retrospective study was done at the Department of Pathology of our institute for a period of two years, from September 2010 to August 2012. Patients with breast lump over this period were included in the study. FNAs were done by a pathologist with all aseptic precautions using 20 mL syringe and 23G needle. The needle was placed within the lesion, and a vacuum was applied by gently withdrawing the plunges of the syringe. The needle was moved back and forth within the lesion, and negative pressure was released prior to withdrawing the needle. Samples were smeared onto glass slides and stained with May-Grunwald-Giemsa (MGG) after being air dried. FNAs over study period were reviewed by two independent cytopathologists. All FNAs of breast masses were classified using criteria similar to NCI guidelines [1]. Cases diagnosed as atypical, probably benign (C3), and suspicious of malignancy (C4) were selected for further study. 

FNA findings of studied smears were then compared with corresponding histopathology findings, obtained with core needle biopsy, excisional biopsy, or mastectomy specimens. Slides of cases of discrepancy were again reviewed, and cytological findings were noted. Based on these findings, sensitivity, specificity, positive predictive values, and negative predictive value of C4 category in diagnosing breast malignancy were calculated. Diagnostic value of cytological diagnosis was assessed by comparing the percentage of benign or malignant histological diagnosis in categories C3 and C4 using chi- square test of significance after Yates correction. 

## 3. Results

During the study period, a total of 1093 FNA cytology of breast lumps were done, out of which 86 (7.8%) had inadequate (C1) smears. Out of 1007 satisfactory FNA smears, 85 (8.43%) were classified in category C3 (*n* = 59, 5.85%) and category C4 (*n* = 26, 2.58%). Of these, 24 cases of C3 category (40.67%) and 16 cases of C4 category (61.53%) had available histologic follow-up data and were included in the study.

Among the C3 category, 9 (37.5%) of C3 cases revealed malignant findings, and 15 (62.5%) of C3 cases revealed benign findings on histologic evaluation (Table 1). Infiltrating ductal carcinoma (*n* = 8) was the most common malignant diagnosis, followed by infiltrating lobular carcinoma (*n* = 1). The commonest benign lesion in C3 category was fibroadenoma (*n* = 8), fibrocystic change with atypia (*n* = 4), and fibrocystic change without atypia (*n* = 3) (Table 2).

Among the C4 category, 14 (87.5%) cases revealed malignant findings, and 2 (12.5%) cases revealed benign findings on final histopathologic analysis (Table 1). All malignant diagnoses were infiltrating ductal carcinoma in category C4. The common benign lesions in category C4 were fibrocystic change with or without atypia (Table 2).

The difference proportion of malignant cases between categories C3 (37.5%) and C4 (87.5%) was statistically significant (*P* = 0.005). Sensitivity, specificity, positive predictive values, and negative predictive value of C4 category in diagnosing breast malignancy were 60.8%, 88.2%, 87.5%, and 62.5%, respectively.

## 4. Discussion

The fine needle aspiration cytology (FNAC) has achieved great importance in diagnosis and management of palpable breast lesions. Due to simplicity, safety, and diagnostic accuracy, this procedure has become a widely used adjuvant diagnostic technique in management of breast lumps [4–6].

The National Cancer Institute recommends five categories for diagnosis of breast aspiration cytology [1] in order to bring a degree of uniformity to the diagnostic reporting. These categories are unsatisfactory (C1), benign lesion (C2), atypical, probably benign (C3), suspicious, probably malignant (C4), and malignant (C5). However, some authors believe that C3 and C4 should be categorized in the same category [2, 3]. In order to investigate this hypothesis further, we reviewed our FNA cases in C3 and C4 and correlated with available histopathological findings to determine the accuracy of C3 and C4 categories. 

Our study had 86 (7.8%) inadequate FNA smears (C1) in concordance with previous reports in the range 0.7–25.3% [7]. So our study found fewer inadequate smears compared with other previous studies because FNA was performed by experienced pathologist of the department. Diagnosis of C2 (benign) was rendered as an adequate sample showing no evidence of significant atypia or malignancy and if representative, a negative report. 

**Figure 1:**
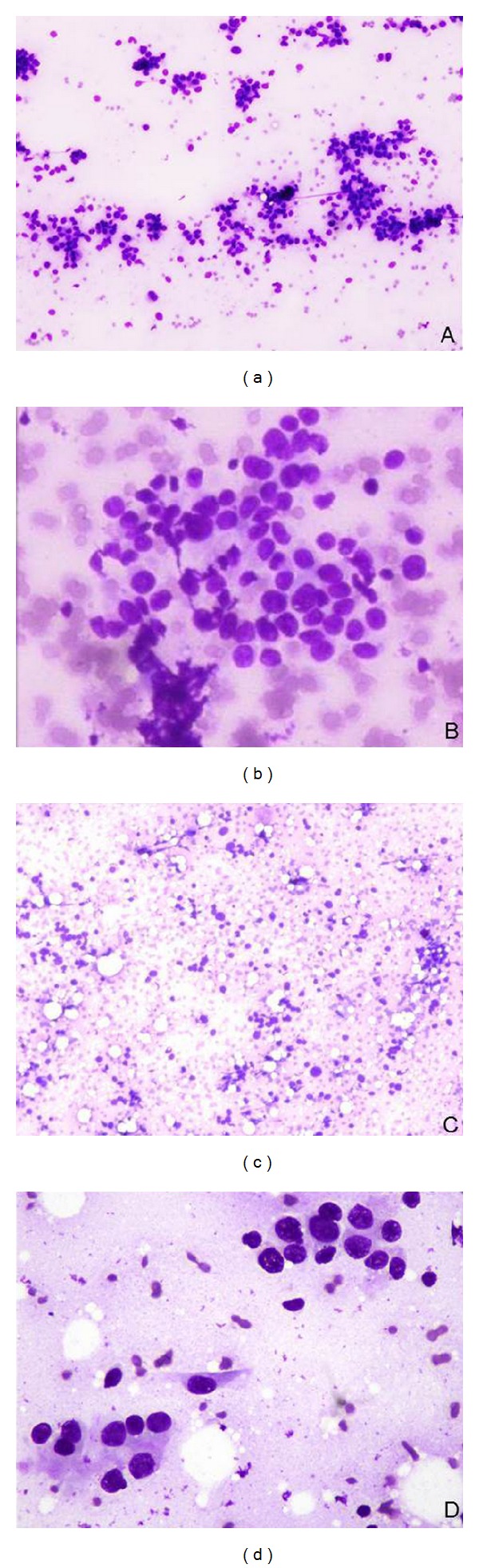
(a) Cases diagnosed as C3 lesion showing slight loss of cohesion of cells (MGG Stain, ×100). (b) Cases diagnosed as C3 lesion showing slight loss of cohesion of cells with mild nuclear atypia (MGG Stain, ×400). (c) Cases diagnosed as C4 lesion showing discohesive cells with moderate nuclear atypia (MGG Stain, ×100). (d) Cases diagnosed as C4 lesion showing loosely cohesive cells with moderate nuclear atypia (MGG Stain, ×400).

We gave cytological diagnosis of atypical, probably benign (C3) when, although the overall pattern of aspirate was benign, there were a population of cells, which showed nuclear pleomorphism, and/or some loss of cellular cohesiveness with nuclear and cytoplasmic changes (Figures 1(a) and 1(b)). A diagnosis that was suspicious of malignancy (C4) usually referred to FNAs that contain cells with some malignant features (Figures 1(c) and 1(d)) in the absence of overtly malignant cells or a aspirate with only scanty number of abnormal cells, or poorly prepared/preserved, but abnormal cells. In our study, based on 1093 cases of breast FNA, the equivocal diagnostic categories C3 and C4 comprised 8.4% of cases. This proportion is in range (4–17.7%) with as reported by others [3, 4, 8–12] indicating that these categories were not underused or overused in our study.

In our study, among the C3 category, 37.5% cases revealed malignant findings on histopathological evaluation (“false negative”). This result also is in concordance with reported literature values where C3 category revealed malignant diagnosis in 8.6–52% cases with most reports having values more than 30% [3, 8, 13]. Infiltrating ductal carcinoma (IDC) was the most common malignant diagnosis of our study followed by lobular carcinoma (Table 2). In C4 category, our study had 87.5% cases with malignant diagnosis, and 12.5% (“false positive”) cases yielded benign diagnosis on histopathological examination. This is similar to other previous studies where 81–97% malignancy was reported in C4 category [3, 8, 13]. 

Factors contributing to “false negative” results include small tumor size, hypocellularity and inadequate sampling during aspiration, few histological tumor types such as low nuclear grade, lobular carcinoma, scirrhous carcinoma, and well-differentiated intra-cystic carcinoma. The most attributed cause of “false negative” rate reported in the literature is sampling error particularly in small tumor [14]. We also underdiagnosed one case of lobular carcinoma as being atypical, probably benign (C3) on FNAC. Review of smear of this case showed few abnormal dissociated cells with slight nuclear atypia against background of benign clusters of epithelial cells and scattered bare nuclei. Majority of “false negative” cases in our study were diagnosed as infiltrating ductal carcinoma on histopathological examination. The cytosmears of these cases showed moderate cellularity with cohesive clusters of epithelial cells. However, on careful review, there were few discohesive clusters with nuclear atypia and nuclear crowding. Presence of naked nuclei in background, lack of discohesion, and apparent monolayered nature of majority of epithelial clusters led to underreporting of these cases as C3 in cytology. 

The two “false positive” diagnoses in our study involved smears that were classified as C4 on cytology but yielded fibrocystic change with or without atypia on histopathological examination. The first case showed mild atypia on histology while the second case showed florid hyperplasia leading to high cellularity and slight loss of cohesion resulting in overdiagnosis on FNAC. In other study also, increased cellularity, loss of cohesion, and nuclear atypia in aspirates from fibroadenoma, fibrocystic disease, and in intraductal papilloma led to erroneous interpretation [15]. In other published reports also, epithelial proliferation of ductal/lobular hyperplasia accounted for “false positive” results [16, 17].

 While the “false positive” results could lead to overtreatment of an unnecessary or excess surgery, “false negative” results can mislead a clinician and cause a delay in treatment. Hence in C3 category, we advise that FNA should not be used as sole modality, and results must be interpreted in correlation with clinical and imaging findings (Triple test) to reduce error and allow proper management of each patient. Our study thus supports that the recommendation of others [9, 18, 19] that use of triple test is important for proper management of patients, with FNA results in C3 category. Similarly, for C4 category with high incidence of malignancy on histopathological examination, we recommend that such patient should definitely have the diagnosis confirmed by histological examination as suggested by other authors also [3, 20].

Thus it is important for both pathologist and clinician to understand not only the benefits but also the limitation of cytological diagnosis from FNA specimen. We feel that it is still useful to maintain the equivocal diagnostic categories C3 and C4 separately, since in our study approximately two thirds of patients who were diagnosed as C3 had benign lesions where about 80% of those in C4 category had malignant diagnosis. This difference was statistically significant (*P* = 0.005). 

## 5. Conclusion

FNAC is simple, safe, cost-effective, and accurate method for initial diagnosis and guiding treatment of breast masses. However, one must be aware of possibility of “false positive” and “false negative” results. Also the category of atypia, probably benign (C3), and suspicious of malignancy (C4) provides a strategy for classification of equivocal cases and allows separation of these cases into clinically useful groups with different probabilities of malignancy and thus different management. 

## Figures and Tables

**Table 1:** Comparison of FNA cytology and histopathology findings.

Cytology category	Histopathology finding		
	Benign lesion	Malignant lesion	Total
C3	15 (62.5%)	9 (37.5%)	24
C4	2 (12.5%)	14 (87.5%)	16

Total	17	23	40

**Table 2:** Histopathology of breast masses according to the cytological diagnosis.

Cytology category	Histopathology finding						
	Benign lesion				Malignant lesion		
	FA	FCC without atypia	FCC with atypia	Total	Invasive ductal carcinoma	Invasive lobular carcinoma	Total
C3	8 (53.3%)	3 (20%)	4 (26.7%)	15	8 (88.9%)	1 (11.1%)	9
C4	0 (0%)	1 (50%)	1 (50%)	2	14 (100%)	0	14

FA: fibroadenoma; FCC: fibrocystic change.
